# Pancreaticopleural Fistula: A Review of Imaging Diagnosis and Early Endoscopic Intervention

**DOI:** 10.1155/2018/7589451

**Published:** 2018-08-19

**Authors:** Ali Kord Valeshabad, Jennifer Acostamadiedo, Lekui Xiao, Winnie Mar, Karen L. Xie

**Affiliations:** ^1^Department of Radiology, University of Illinois at Chicago, USA; ^2^Division of Interventional Radiology, University of Illinois at Chicago, USA; ^3^Department of Medicine, Mercy Hospital and Medical Center, Chicago, IL, USA

## Abstract

A 49-year-old male with history of chronic alcohol-induced pancreatitis presented with one month of worsening left pleuritic chest pain and shortness of breath. Chest radiograph demonstrated bilateral pleural effusions. Thoracentesis revealed increased amylase in the pleural fluid. Computed tomography (CT) and magnetic resonance cholangiopancreatography (MRCP) showed a fistula tract between the left pleural cavity and pancreas which was confirmed on endoscopic retrograde cholangiopancreatography (ERCP). Patient was treated with placement of a pancreatic stent with complete resolution of the fistula tract approximately in 9 weeks. A systematic literature search was performed on reported cases with pancreaticopleural fistula (PPF) who underwent early therapeutic endoscopy within the last 10 years. Imaging modalities, particularly CT and MRCP, play essential role in prompt preprocedural diagnosis of PPF. Early therapeutic ERCP is an effective and relatively safe treatment option for PPF, so invasive surgery may be avoided.

## 1. Introduction

Pancreaticopleural fistula (PPF) is an uncommon complication of chronic pancreatitis, often presenting with recurrent unilateral or bilateral pleural effusions [1, 2]. There is usually an abnormal communication to the pleural space from posterior pancreatic duct disruption or pancreatic pseudocyst extension into the pleural cavity [3]. Minimally invasive endoscopic intervention following medical therapy is usually the first choice before invasive surgical management [2, 3]. To our knowledge, there is no recent comprehensive data available regarding the outcome of early endoscopic treatment in the patients with PPF. In this study, we present a case of PPF treated successfully by endoscopic retrograde cholangiopancreatography (ERCP) and transpapillary pancreatic duct stent placement. In our case imaging played an important role in the investigation of PPF and follow-up after ERCP. We performed a systematic literature search of reported PPF cases who underwent early therapeutic endoscopy within the last 10 years, with a review of the utility of diagnostic imaging and the outcome of endoscopic management of PPF.

## 2. Methods

A review of the literature was performed using the MEDLINE database with searching of the keywords: pancreaticopleural fistula, pancreatico-pleural fistula, and pancreaticopleural effusion. Inclusion was limited to cases reported in the English or Spanish language between 2007 and 2017 who underwent early ERCP alone or before any operative management. Forty-three patients (including the patient in this study) with PPF were reported during the 2007-2017 period [2, 4–36]. Data is presented as percentage or mean values ± standard deviation (SD). N throughout the manuscript stands for total number of cases with available data for that variable.

## 3. Results

### 3.1. Case Presentation

**Figure 1:**
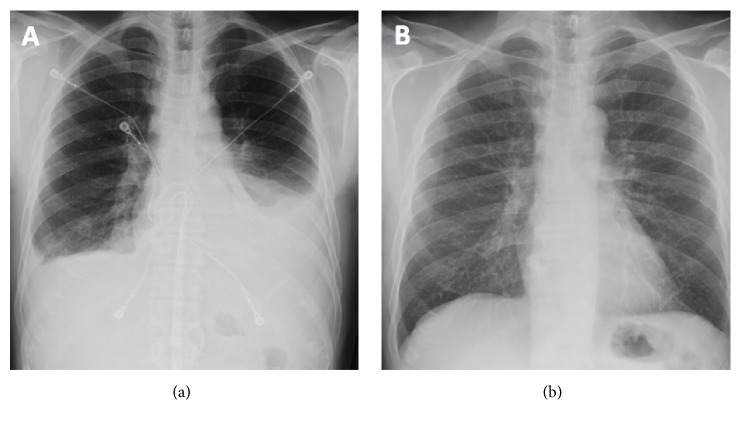
Chest radiograph at admission (a) demonstrates moderate left and trace right pleural effusions with a left retrocardiac opacity. Follow-up chest radiograph shows complete resolution of the abnormalities after treatment (b).

A 49-year-old male with history of alcohol-induced pancreatitis presented with 1 month of worsening left pleuritic chest pain and shortness of breath. Initial physical exam was unremarkable, and laboratory tests were only remarkable for increased lactate dehydrogenase and borderline-low albumin. Initial chest radiograph showed moderate left and small right pleural effusions with left retrocardiac opacity (Figure 1). Computed tomography (CT) pulmonary angiogram was performed and was negative for pulmonary embolism and showed only small bilateral pleural effusions.

**Figure 2:**
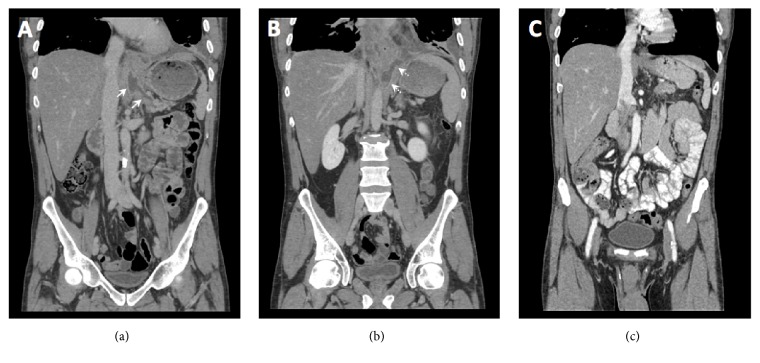
Coronal views of contrast-enhanced CT of the abdomen and pelvis show a fistula tract arising from the tail of the pancreas ((a) white arrows) extending caudally and connecting to the left pleural space ((b) dashed white arrows) with small pleural effusion and overlying atelectasis. A follow-up CT (c) demonstrates resolution of the fistula tract and pleural effusion.

**Figure 3:**
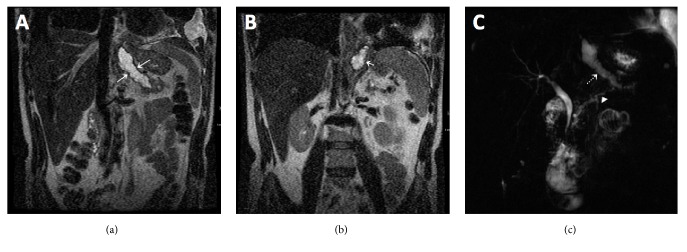
Coronal T2-weighted MRCP images (a, b) demonstrate a hyperintense tract arising from the tail of pancreas ((a) large white arrows), crossing the diaphragm and entering the pleural space ((b) short white arrow). Thick slab 2D MRCP images (c) show an irregular main pancreatic duct (white arrow head) and redemonstrates the fistula tract arising from the tail of pancreas (white dashed arrow).

On admission, patient underwent thoracentesis and approximately 1L of brown fluid from the left pleural space was removed. Pleural fluid evaluation demonstrated an exudative effusion with lipase of 2912 IU/L and amylase of 2783 IU/L. CT abdomen and pelvis demonstrated a fluid density tract arising from the pancreatic tail, extending caudally through the diaphragm from the esophageal hiatus, communicating with the left pleural space (Figure 2). The patient was started on medical therapy with octreotide. Magnetic resonance cholangiopancreatography (MRCP) confirmed a PPF tract with irregular, dilated, main pancreatic and common bile ducts (Figure 3). The patient underwent ERCP two days after admission which redemonstrated mild diffuse dilatation of ventral pancreatic duct involving the head, body, and tail of the pancreas with evidence of pancreatic duct leak in the most upstream tail of the pancreas that extended caudally to the left pleural cavity, consistent with PPF. A 5 FR x 10 cm plastic stent was placed in the pancreatic duct and a biliary and pancreatic sphincterotomy was performed to divert the fluid leak.

The hospital course was complicated by post-ERCP acute pancreatitis, which was treated with bowel rest and empiric antibiotics. Postprocedure chest radiograph showed worsening pleural effusions and surgical management including distal pancreatectomy was considered. However, surgery was deferred after the patient improved clinically with medical management, and a repeat chest radiograph revealed improvement of the pleural effusions. The patient improved clinically and was discharged with pain medications.

The patient returned to the emergency department within the first week after discharge with complaints of worsening abdominal and chest pain. Another thoracentesis was performed with removal of 400 mL fluid. The study of pleural fluid specimen demonstrated amylase level > 1000 IU/L which was positive for Klebsiella and enterococcus faecalis. The patient was treated with intravenous antibiotics and the pancreatic stent was upsized to 7Fr.

**Figure 4:**
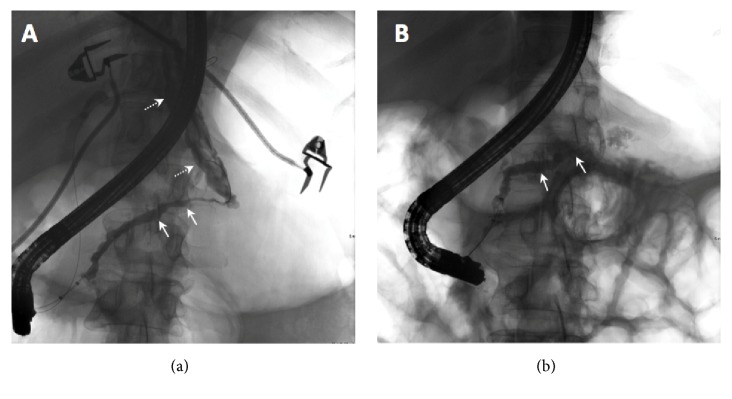
ERCP at admission (a) shows mild diffuse dilatation of ventral pancreatic duct involving the head, body, and tail of the pancreas (white arrows) with pancreatic duct leak in the most upstream tail of the pancreas that extends caudally to the pleural cavity (dashed white arrows). A follow-up ERCP after treatment (b) demonstrates mild diffuse dilatation of the main pancreatic duct with resolution of the fistula tract.

Approximately 9 weeks following the initial ERCP, repeat ERCP demonstrated no extravasation of contrast from the pancreatic tail, and the stent was downsized to 5Fr. Repeat ERCP 15 weeks after the index procedure showed mild diffuse dilatation of the main pancreatic duct with no extravasation of contrast or stenosis (Figure 4). Therefore, the pancreatic duct stent was removed. Chest radiograph and CT abdomen pelvis showed complete resolution of pleural effusions bilateral as well as the PPF tract (Figures 1 and 2).

### 3.2. Previous Cases of Pancreaticopleural Fistula

Table 1 summarizes the demographics and clinical data of previously reported cases of PPF. Seventy-nine percent of the cases were male with a mean age of 50 ± 13 years (N = 41, range: 5-78 years). History of alcohol use and chronic pancreatitis was present in 78% (N = 40) and 67% (N = 30) of the patients, respectively. All patients presented with respiratory symptoms including shortness of breath and pleuritic chest pain. The pleural fluid amylase level ranged 1200-138000 IU/L (N = 43).

Chest radiograph was performed in 95% of the patients and showed unilateral or bilateral pleural effusion. Forty (93%) of the patients underwent CT which demonstrated the fistula tract in 25 out of 40 patients (63%). MRCP was performed in 24 patients which revealed the fistula tract in 20 of them (83%). The fistula tract was visualized in 30 patients out of 43 (70%) during the ERCP procedure. In 3 patients the diagnosis was made based on the high amylase level of the pleural fluid.

The majority of the patients (85%, N = 39) received medical therapy before any intervention. The medical therapy included octreotide, somatostatin, total parenteral nutrition, and antibiotics. The time to ERCP after admission was available only in 4 cases and ranged 1-21 days. Post-ERCP acute pancreatitis was reported in 9.5% of cases (N = 21) and superinfection of the pleural fluid occurred in 27.0% of patients (N = 22). Twenty-three out of 43 patients required surgery after the initial ERCP, including distal pancreatectomy (30%), surgical thoracostomy/decortication (30%), pancreaticojejunostomy (18%), exploratory laparoscopy, and external drainage (18%). One patient (4%) underwent surgical sphincterotomy secondary to difficult anatomy for ERCP. ERCP alone and ERCP with surgery were successful in 100% and 96% of patients, respectively. The total duration of the hospital stay was 27 ± 16 days (N = 28). One patient (2%, N = 43) with refractory pleural effusion refused surgical intervention and died.

## 4. Discussion

### 4.1. Demographics and Presentations

Pancreatic Pleural Fistula (PPF) is a rare entity with an approximate incident rate of 0.4%. Based on this literature review, PPF usually involves males in their late 40s with chronic pancreatitis mainly from excessive alcohol abuse, consistent with previous studies [1]. A smaller percentage of cases may be due to biliary stones, trauma, and idiopathic pancreatitis [1]. Patients with PPF usually present to the emergency room with respiratory symptoms including shortness of breath [37], as seen in our patient, and less commonly with diffuse upper quadrant abdominal pain, nausea, and vomiting without rebound or guarding [1, 2, 8, 27].

### 4.2. Diagnosis

History and physical examination are nonspecific for PPF and further evaluation with imaging and laboratory work-up is required. In patients with PPF, chest radiograph usually demonstrates unilateral or bilateral pleural effusion, with the left side being more common. Blood laboratory evaluation may show increased levels of gamma-glutamyl transferase [37]. Thoracentesis is usually required for symptom relief. Laboratory analysis of the pleural fluid shows an amylase level greater than 1000 IU which may be suggestive for PPF in the absence of malignant cells [1]. In our patient, the initial CT imaging showed the fistula communication between the tail of the pancreas and left pleural space which was confirmed by MRCP and ERCP. From our review, CT was performed on majority of patients with PPF (93%) and was able to identify the fistula in 63% of patients which was comparable with ERCP (70%). Compared to ERCP, CT has the advantages of being noninvasive and relatively less expensive and can be completed in a much shorter time with no sedation required. ERCP has the advantages that it can locate the site of ductal leak and perform simultaneous intervention [2, 3, 30, 37]. In old studies CT was reported to identify fistula tract only in 30% of cases with PPF [1]; application of high resolution CT images with multiplanar reconstruction has likely increased the ability to visualize the fistula tract in newer studies. MRCP showed fistula tract in 83% of patients with PPF in this study, which is similar to reported studies [38]. In addition, CT and MRCP are useful in evaluating additional pancreatic parenchyma and ductal structural abnormalities such as pancreatic mass, pseudocysts, or collections [32, 35], which is valuable for surgical or interventional planning [1].

### 4.3. Management and Outcome

The majority of patients (85%) in the studies we reviewed received medical therapy before any intervention, consistent with previous reports [1]. The medical therapy included octreotide, somatostatin, total parenteral nutrition, and antibiotics. King et al. [1] reported the outcome of early operative management in 63 patients with PPF between 1970 and 2008. In their study, the early operative management had a success rate of 94%. Six patients underwent ERCP which was successful in all cases. ERCP alone and ERCP with surgery was successful in 100% and 96% of patients in our review, which is comparable to king et al. study. The total duration of the hospital stay in this review was 27 ± 16 days which was relatively shorter than reported 40 ± 6 days for patients who received medical therapy and surgery [1]. Postprocedural acute pancreatitis and superinfection of the pleural fluid were the most common complications after ERCP based on our review. Endoscopic pancreatico- or cystogastric plastic stent placement may be helpful to avoid postprocedural acute pancreatitis. Some other less common complications are intra-abdominal infections, wound infections, entrapped lung and in some cases diabetes mellitus after surgery [2]. Only one patient (2%) died after the index endoscopic procedure who had refractory pleural effusion and refused surgical intervention. The mortality rate of patients with PPF who underwent surgical intervention with or without medical therapy has been reported as high as 12-17% [1, 3]. In addition, in patients after ERCP ductal stenting there is a faster transition to feeding and reduction of recovery time. ERCP is both a diagnostic and therapeutic tool which has reduced the hospital stay and mortality rate compared to the traditional operative management [2–4, 37], although further prospective studies are still required to compare the cost-effectiveness and long-term outcome of ERCP with surgery.

## 5. Conclusion

Imaging modalities, particularly CT and MRCP, play essential role in prompt preprocedural diagnosis of PPF. Early therapeutic ERCP is an effective and relatively safe treatment option for PPF, so invasive surgery may be avoided.

## Figures and Tables

**Table 1:** Demographics and clinical data of previously reported cases of pancreaticopleural fistula between 2007 and 2017. N = total number of cases. n is the number of cases data reported. SD = standard deviation.

**Variables**	**N**	n (percentage) or Mean ± SD
Male gender	43	34 (79%)
Mean Age (years)	41	50 ± 13
History of chronic pancreatitis	30	20 (47%)
History of alcohol use	40	31 (78%)
Pleural fluid amylase (IU/L)	43	30021 ± 25410
CT Identified Fistula Tract	40	25 (63%)
MRCP Identified Fistula Tract	24	20 (83%)
ERCP Identified Fistula Tract	43	30 (70%)
Medical treatment	39	33 (85%)
Post-ERCP acute pancreatitis	21	2 (9.5%)
Post-ERCP superinfection of pleural fluid	22	6 (27.0%)
Operative management	43	23 (53%)
Partial/total pancreatectomy		7 (30%)
Thoracostomy/decortication		7 (30%)
Pancreaticojejunostomy		4 (18%)
Exploratory laparoscopy and external drainage		4 (18%)
Surgical sphincterotomy		1 (4%)
Duration of hospital stay (days)		27 ± 16
Mortality	43	1 (2%)
